# Eversion congénitale bilatérale des paupières: prise en charge d’un cas selon l’approche conservatrice au Centre Hospitalier Universitaire de Yaoundé, Cameroun

**Published:** 2012-02-28

**Authors:** Francisca Monebenimp, Gilles Kagmeni, David Chelo, Yannick Bilong, Ernest Moukouri

**Affiliations:** 1 Département de Pédiatrie, Faculté de Médecine et des Sciences Biomédicales, Université de Yaoundé I, Cameroun; 2 Centre Hospitalier et Universitaire de Yaoundé, Cameroun; 3 Département d’Ophtalmologie et ORL, Faculté de Médecine et des Sciences Biomédicales, Université de Yaoundé I, Cameroun; 4 Centre Mère et Enfant de la Fondation Chantal Biya, Yaoundé, Cameroun

**Keywords:** Eversion palpébrale, congénitale, nouveau-né, traitement conservateur, ponction, Cameroun

## Abstract

L’éversion congénitale des paupières est une affection rare. Son traitement en première intention est généralement conservateur, constitué de lubrifiant, d’antibiotiques, de manœuvres d’inversion de la paupière éversée et d’une éducation des parents. Nous présentons le cas d’un nouveau-né de huit heures de vie ayant une éversion congénitale bilatérale des paupières avec surinfection bactérienne. La ponction à l’aiguille de la conjonctive œdémateuse associée au traitement topique avec du sérum salé isotonique et des antibiotiques ont accéléré le processus de guérison. Une récidive n’a pas été observée lors des pleurs après trois semaines d’inversion des paupières.

## Introduction

L’éversion congénitale de la paupière est définie comme une extériorisation de la conjonctive palpébrale, le plus souvent constatée à la naissance mais pouvant aussi se révéler tardivement [1,2]. Il s’agit d’une affection rare, qui peut être isolée ou associée à d’autres anomalies ou syndromes malformatifs [2,3]. L’évolution naturelle est marquée par la survenue de l’ophtalmie néonatale, des lésions cornéennes, ou d’une inversion palpébrale instable chronique [1,4,5]. La prise en charge est conservatrice et selon le cas elle peut être chirurgicale. Nous présentons une approche conservatrice de prise en charge d’un nouveau-né ayant un chémosis bilatéral associé à un écoulement purulent.

## Observation

Il s’agit d’un nouveau-né de huit heures de vie, de sexe masculin, vu en consultation d’ophtalmologie pour une malformation oculaire constatée à la naissance.

Sa mère âgée de 33 ans, a eu une grossesse bien suivie. Les sérologies de la syphilis et du VIH réalisées au 2^ème^ trimestre de la grossesse étaient négatives. Les sérologies, toxoplasmose et rubéole faites au 3^ème^ trimestre de grossesse étaient par contre positives avec des taux respectifs d’Ig G de 15 UI/ml et de 100 UI/ml. Aucune information n’était connue sur le prélèvement cervico vaginal. C’était une septième grossesse à terme sans notion de rupture prématurée ou prolongée des membranes, de liquide amniotique méconial ou purulent ni de fièvre maternelle en péripartum. La durée de travail était de six heures. L’accouchement était eutocique, par voie basse et la présentation céphalique. Le score d’Apgar était de 10 à la 1^ère^ et 5^ème^ minute. Le père âgé de 45 ans, n’avait aucun antécédent médical particulier. Par ailleurs, Il n’y aurait pas de notion de malformation congénitale apparente dans les deux familles parentales.

**Figure 1:**
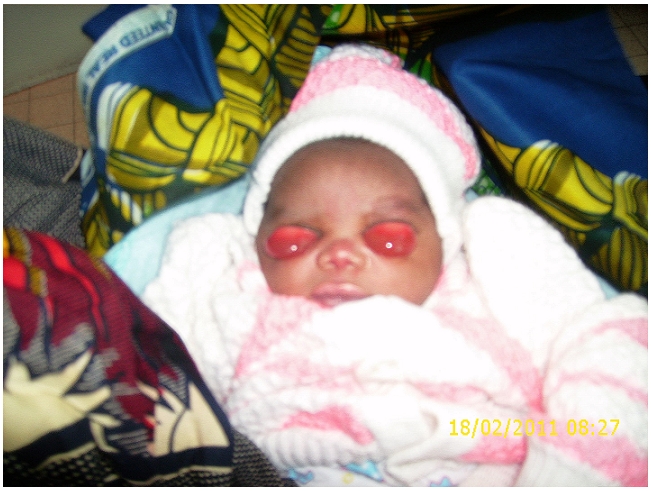
Eversion palpébrale à l’admission. vue de face

**Figure 2:**
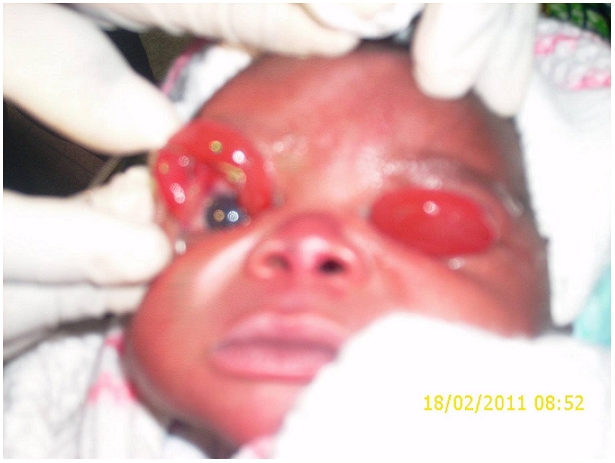
Eversion palpébrale à l’admission. pose du blépharostat, globe oculaire présent et anatomiquement sain

A l’examen ophtalmologique du nouveau-né, on notait une éversion totale des deux paupières supérieures. Il y avait également une tuméfaction des conjonctives palpébrales septales avec une transillumination positive (Figure 1). Les conjonctives hyperhémiées laissaient sourdre des secrétions épaisses jaunâtres. Après lavage au sérum physiologique et instillation d’un collyre anesthésique local, la pose d’un blépharostat avait fait objectiver une morphologie normale des globes oculaires (Figure 2).

Le reste de l’examen clinique effectué par le pédiatre, ne révélait aucune autre anomalie apparente. Sur le plan anthropométrique, le nouveau-né était situé au 75^ème^ percentile pour le poids et la taille. Il était stable sur le plan hémodynamique et avait une température de 37,1°C.

Le diagnostic retenu était celui d’une éversion congénitale bilatérale isolée des paupières supérieures associée à une ophtalmie néonatale.

Les examens complémentaires suivants ont été réalisés: 1) L’examen cytobactériologique des sécrétions conjonctivales qui a révélé la présence de nombreux leucocytes et de deux germes : *Escherichia coli* et *Staphylococcus aureus* sensibles à la Ceftriaxone et la Gentamycine ; 2) Echographie oculo-palpébrale en mode B qui a objectivé une collection liquidienne exclusivement sous-palpébrale, septale, diffuse et hétérogène évoquant un chémosis. Les globes oculaires avaient des mensurations proportionnelles à l’âge et une structure normale ; 3) L’hémoculture et la protéine C-réactive visant à exclure une infection néonatale systémique se sont avérées non contributives.

Le nouveau-né hospitalisé dans l’unité de néonatologie a eu un lavage oculaire bilatéral au sérum salé isotonique toutes les quatre heures et ceci jusqu’à l’arrêt des secrétions purulentes puis une application locale de la Tétracycline pommade à 1% toutes les 12 heures durant l’hospitalisation. Il est à noter que les paupières étaient recouvertes à chaque fois d’une compresse stérile reposant sur du tulle gras. La compresse était maintenue sans compression sur deux coins par du ruban adhésif. Sur le plan parentéral, une antibiothérapie probabiliste à base de Ceftriaxone et de Gentamycine avait été instaurée et ceci pendant 10 jours au regard de la normalité de la numération formule sanguine et des CRP de contrôle réalisés.

**Figure 3:**
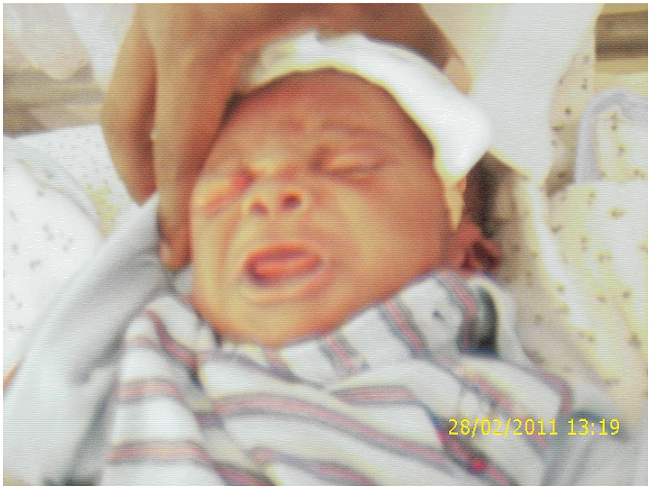
Après les ponctions conjonctivales. J10 d’hospitalisation: réduction du chémosis avec éversion lors des pleurs

**Figure 4:**
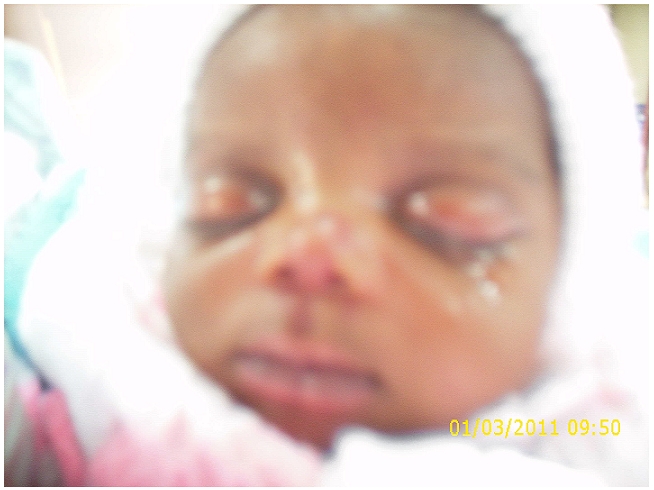
Après les ponctions conjonctivales. J11 d’hospitalisation: Paupières éversées par la mère en absence des pleurs avec un chémosis résiduel

Au 3^ème^ jour d’hospitalisation une ponction aspiration du chémosis à l’aide d’une aiguille 23G effectuée à deux reprises du côté droit a été blanche. Aux points de piqûre suintait une sérosité fluide de couleur jaune citrin imbibant la compresse. Après un délai de 48 heures, la réduction importante du chémosis à droite a encouragé la réalisation du même geste du côté opposé. Entre le 10 et le 11^ème^ jour de suivi, il était associé au chémosis résiduel, un pseudo-ptosis et une éversion palpébrale transitoire, dans la mesure où il y avait récidive lors des pleurs (Figure 3 et Figure 4). Une traction en avant de la paupière supérieure montrait un décollement du globe sur plus de 8 mm signant une hyperlaxité. Le renforcement des capacités de la mère a consisté en la démonstration des techniques de lavage des mains et d’inversion délicate des paupières en cas de récidive. La sortie du nouveau-né a été autorisée après interruption des topiques locaux au 11^ème^ jour. Un rendez-vous pédiatrique à deux semaines puis à un mois de vie pour l’ophtalmologie ont été donnés à la mère.

A la visite d’un mois de vie, la mère rapportait que les récidives de l’éversion bilatérales s’étaient amandées dès la 3^ème^ semaine. L’examen n’objectivait aucune hyperlaxité ni de ptose palpébrale. La statique et la dynamique palpébrale étaient normales.

## Discussion

L’éversion congénitale de la paupière se présente comme une exposition totale de la conjonctive tarsale, associée à une inflammation et un chémosis d’intensité variable [1,5,6]. Le nouveau-né vu avait une éversion congénitale bilatérale, symétrique de la paupière supérieure. Bien que la plupart du temps les deux paupières supérieures soient affectées de façon symétrique, il existe des formes unilatérales [5].

Il s’agit d’une pathologie rare, dont l’incidence serait plus importante au sein de la race noire et au cours de certaines anomalies comme la trisomie 21 et l’ichtyose [2,3]. La présence de telles affections évoquent des formes associées ou secondaires. Ceci n’était pas le cas chez notre patient qui avait une forme isolée ou primaire. En effet, aucune embryo- foetopathie, ni autre anomalie apparente n’ont été mises en évidence à l’examen physique.

La physiopathologie exacte de l’éversion palpébrale est peu élucidée. La parité, la durée du travail et le mode d’accouchement sont des facteurs prédisposant contestés [5]. Parmi les hypothèses évoquées, on peut retenir la théorie du spasme du muscle orbiculaire associée ou non à la présence de prédispositions anatomiques palpébrales favorisant l’éversion [1]. Ces spasmes du muscle orbiculaire entraineraient une entrave au retour veineux, dont la résultante serait un chémosis qui par action mécanique induirait l’éversion de la paupière. Par ailleurs, ces spasmes peuvent être induits par une irritation conjonctivale, une obstruction du retour veineux palpébral, voire un traumatisme facial occasionné par des contractions utérines ou le passage dans la filière génitale [7–10]. Ce chémosis, représente l’élément diagnostic différentiel capital distinguant l’éversion de l’ectropion congénital palpébral (Tableau 1). La possibilité d’un kyste conjonctival bilatéral et congénital a été exclu grâce à l’absence d’une paroi limitant la collection liquidienne sous palpébrale à l’échographie oculaire.

**Tableau 1:** Différences cliniques entre l’éversion et l’ectropion congénital de la paupière

Critères	Eversion palpébrale congénitale	Ectropion palpébral congénital
Définition	extériorisation de la conjonctive palpébrale.	Bascule en dehors du bord libre de la paupière.
Siège	Plus souvent la paupière supérieure [1,5,6]	Plus souvent la paupière inférieure [16]
Physiopathologie	Spasmes de l’orbiculaire associés ou non à des prédispositions anatomiques à l’éversion de la paupière [1,5,6].	les anomalies anatomiques sont constants et responsables de la pathologie : hypotonie de la sangle tarso-tendineuse associée à un trouble du système rétracteur avec atteinte de son faisceau postérieur et à une brièveté de type cicatriciel de la lamelle antérieure avec hypoplasie de l’orbiculaire pré-tarsal. [16]
Signes cliniques	- La base d’implantation des cils n’est pas en contact avec le globe oculaire ;	
	- Constat à la naissance ;	
	- Fréquemment associés aux anomalies congénitales : ichtyose, trisomie 21 [1,5,6].	
	**Chémosis présent**	**Absence de chémosis**
Complications	- Kératinisation de la conjonctive [1]	
	- Ophtalmie néonatale pouvant être associée à une infection néonatale [5]	
	- Rare atteinte cornéenne [4]	- Atteinte cornéenne fréquente [4]
	- Instabilité palpébrale (Résolution spontanée possible avec récidives récurrentes lors des pleurs) [1]	- le plus souvent persistance en absence de chirurgie [1]
Traitement	- Conservateur en 1^ère^ intention	Chirurgical d’emblée [1,15,17]
	- Chirurgical en 2^ème^ intention [1,5,6]	

Ce nouveau-né aurait eu une irritation conjonctivale consécutive à une inflammation causée par l’infection à *Escherichia Coli* et à *Staphylococcus aureus* in utéro. Au regard de la précocité de survenue de l’ophtalmie néonatale associée à l’éversion palpébrale (moins de 24h de vie) et le temps d’incubation habituel des germes isolées d’environ quatre à cinq jours [11], nous pouvons dire que le processus infectieux aurait débuté en antépartum. Pour cela, nous ne retrouvions aucun argument anamnestique étayant un risque infectieux à l’histoire obstétricale bien qu’aucun prélèvement cervico-vaginal n’ait été fait durant la grossesse et après la délivrance pour corroborer cette hypothèse. Adeoti et al en 2010 avaient rapporté un cas d’infection néonatale précoce à 24h de vie associée à une ophtalmie néonatale sur éversion palpébrale congénitale [5].

Plusieurs prédispositions à une éversion palpébrale supérieure ont été décrites: l’hypotonie du muscle orbiculaire, la laxité de l’union des lamelles antérieure et postérieure [12], les traumatismes palpébraux à la naissance, la brièveté de la lamelle antérieure ou l’élongation verticale de la lamelle postérieure de la paupière, l’absence d’insertion du septum orbitaire à l’aponévrose de l’élévateur de la paupière supérieure [13], la laxité du ligament canthal interne et l’élongation latérale de la paupière [2].

Le traitement de l’éversion congénitale de la paupière a été conservateur. Il avait pour buts : d’une part de prévenir la déshydratation de la conjonctive exposée, par l’instillation du sérum physiologique et l’application de la pommade ophtalmique; et d’autre part de favoriser l’inversion permanente de la paupière éversée, par des manœuvres correctrices dés la résorption du chémosis [1,5–7].

Chez ce nouveau-né, la ponction à l’aiguille des conjonctives a permis la résorption du chémosis grâce au suintement des sérosités. Cette procédure mécanique de prise en charge d’un œdème a été similaire à celle utilisée en acupuncture [14]. Habituellement l’on a recourt à une solution salée hypertonique à 5 % agissant par osmose avec un délai thérapeutique de 7 à 10 jours [5,6] tandis que le délai de réduction du chémosis après ponction à l’aiguille était de 48 heures chez notre patient.

D’autres alternatives plus invasives du traitement de l’éversion palpébrale congénitale existent notamment : la tarsorraphie temporaire, la résection des bords latéraux des paupières supérieure et inférieure, la tarsectomie avec mullarectomie, les greffes de peau, l’excision de la conjonctive en excès, le rétablissement de l’union entre les lamelles antérieure et postérieure grâce à la reconstruction des fornix, et les injections sous-conjonctivales de hyaluronidase [12,15].

## Conclusion

L’éversion congénitale de la paupière est une affection rare. La ponction à l’aiguille de la conjonctive associée aux moyens conservateurs classiques a permis de raccourcir le délai de résorption du chémosis et d’accélérer le processus de guérison. La collaboration de l’ophtalmologue et du pédiatre reste indispensable pour une prise en charge efficace du nouveau-né.
